# Evaluating oxygen reserve index-guided oxygenation for the prevention of postoperative delirium in elderly patients: a randomized controlled trial

**DOI:** 10.3325/cmj.2025.66.47

**Published:** 2025-02

**Authors:** Pelin Uzun Sarıtaş, Aykut Sarıtaş, Merve Çetin Poyraz, Uğur Uzun

**Affiliations:** 1 Department of Anesthesiology and Reanimation, İzmir City Hospital, İzmir, Turkey; 2 Department of Anesthesiology and Reanimation, Health Sciences University, Tepecik Training and Research Hospital, İzmir, Turkey

## Abstract

**Aim:**

To assess the effect of oxygen reserve index (ORi)-guided titration of fraction of inspired oxygen (FiO_2_) on the incidence of postoperative delirium (POD) and the frequency of hyperoxemia episodes, assuming a potential link between hyperoxemia and POD.

**Methods:**

This randomized controlled trial included 114 patients aged 65 years and older, scheduled for elective surgeries lasting over two hours at Health Sciences University Tepecik Training and Research Hospital between October 1, 2023, and July 7, 2024. Patients were randomized into the control group (n = 57) or the ORi + pulse oximetry (SpO_2_) group (n = 57). In the ORi+SpO_2_ group, we dynamically adjusted FiO_2_ based on the real-time ORi and SpO_2_ values, targeting 95%<SpO_2_≤98% and ORi 0.00. In the control group, FiO_2_ adjustments were guided solely by SpO_2_ readings, with ORi values recorded but not used for decision-making. POD was assessed with the Confusion Assessment Method (CAM) and CAM-ICU at multiple time points over 48 hours.

**Results:**

The incidence of POD was significantly higher in the control group than in the ORi+SpO_2_ group (42.1% vs 12.3%, *P* < 0.001). The average FiO_2_ levels in the ORi+SpO_2_ group were significantly lower throughout surgery (*P* < 0.001). The ORi+SpO_2_ group also exhibited a lower frequency of hyperoxemia episodes.

**Conclusion:**

The ORi-guided oxygenation strategy significantly reduced the incidence of POD in elderly patients by effectively minimizing intraoperative hyperoxemia. Optimizing intraoperative oxygenation through non-invasive monitoring may enhance perioperative outcomes.

Clinicaltrials.gov registration number: NCT06326372

Postoperative delirium (POD) is a common and serious complication affecting elderly patients undergoing surgical procedures (1). It is an acute neurocognitive disorder characterized by cognitive decline, fluctuating mental status, impaired consciousness, inattention, and confusion. A considerable percentage of elderly patients admitted to hospitals experience POD, which makes it a particularly concerning condition in this population (2-4). The incidence rates among patients undergoing cardiac surgery reach as high as 50%-67%, while the rates for certain types of surgeries amount to 72% (5-7).

The consequences of POD include increased morbidity and mortality, extended hospital stays, delayed physical recovery and rehabilitation, permanent cognitive decline, and higher health care costs. The treatment and management of POD are challenging, necessitating a comprehensive approach (1,8-10).

The etiology of POD involves a wide range of predisposing and precipitating factors (10,11). While significant attention has been given to hypoxia as a contributing factor, the role of intraoperative oxygenation strategies remains less well explored. Intraoperative hyperoxemia, defined as an excess of oxygen in body tissues during surgery, has been associated with systemic vasoconstriction, increased oxidative stress, and inflammation, all of which could contribute to neurocognitive dysfunction (12,13). These physiological effects may be particularly relevant in elderly surgical patients, who are more vulnerable to such stresses.

Intraoperative hyperoxemia is frequently observed in clinical practice, yet it is often left unmonitored or untreated. Numerous studies have linked hyperoxemia to increased mortality, but data regarding its impact on postoperative neurological outcomes remain inconsistent (12-16). As preclinical evidence suggests an association between intraoperative hyperoxemia and poor neurological outcomes, the risks associated with excessive oxygen exposure may be mitigated by optimized oxygen administration.

The oxygen reserve index (ORi) is a non-invasive monitoring tool that provides real-time feedback on the patient's oxygenation status, allowing for more precise titration of inspired oxygen (FiO_2_) levels. By optimizing intraoperative FiO_2_ delivery, ORi-guided oxygenation strategies may help minimize excessive oxygen exposure while maintaining adequate oxygenation. While previous studies have explored the effects of intraoperative oxygenation strategies, there is a lack of randomized controlled trials specifically investigating the impact of ORi-guided FiO_2_ titration on POD incidence. The primary aim of this study was to evaluate whether ORi-guided FiO_2_ titration reduces the incidence of POD in elderly patients. The secondary aim was to determine whether ORi-guided oxygenation reduces the occurrence of intraoperative hyperoxemia episodes.

## Patients and methods

### Study design

This randomized controlled study was conducted in accordance with the principles of the Declaration of Helsinki. It was approved by the Institutional Review Board of the Health Sciences University İzmir Tepecik Training and Research Hospital. The patients voluntarily participated in the study and provided informed consent.

### Participants

A total of 122 patients aged 65 and older were initially assessed for eligibility at Health Sciences University Tepecik Training and Research Hospital. Six patients were excluded due to a shorter-than-expected duration of surgery and 2 due to a different anesthesia regimen, which left 114 patients in the final sample.

The patients had to have the American Society of Anesthesiologists (ASA) scores of 1-4, be scheduled for elective surgery lasting more than two hours, and be scheduled for postoperative monitoring at least two days.

The non-inclusion criteria were emergency surgery, a preoperative Mini-Mental State Examination (MMSE) score of 23 or lower, known dementia or central nervous system diseases, brain or cardiovascular surgery, inability to communicate, and not consenting to participate.

### Randomization

Patients were randomly assigned to the control group (n = 57) or the ORi+SpO_2_ group (n = 57). The study statistician generated random numbers using an online randomization system (https://randomizer.org) without any restrictions. These random numbers were placed in sequentially numbered envelopes and provided to a research coordinator the day before surgery by a research nurse.

The research coordinator disclosed the group assignments to the anesthesiologist after obtaining consent from the participants. The anesthesiologist, aware of the group allocations, adjusted the FiO_2_ levels accordingly for each participant in the ORi+SpO_2_ group. The control group was only observed, and data were recorded without any intervention. The anesthesiologist could not be blinded to the group assignments due to the necessity of knowing the FiO_2_ targets for proper adjustment. However, the outcome assessments and statistical analyses were conducted independently by researchers who were blinded to the group assignments. The surgical team and researchers assessing the outcomes were also blinded to group assignments.

### Preoperative evaluation

In the preoperative evaluation room, patients underwent MMSE. We also recorded data on demographic characteristics, comorbidities, ASA scores, and smoking status. Upon arrival in the operating room, routine anesthesia monitoring was performed.

### Charlson Comorbidity Index calculation

The Charlson Comorbidity Index (CCI) was calculated to assess the comorbidity burden. The standard CCI was used without age adjustment. Each comorbid condition was assigned a weighted score according to the original Charlson methodology. The major comorbid conditions observed included diabetes, cardiovascular disease, chronic obstructive pulmonary disease, myocardial infarction, dementia, and cerebrovascular disease.

For interpretability, CCI scores were categorized into four clinically meaningful groups: 0: no comorbidities present; 1-2: mild comorbidity; 3-4: moderate comorbidity; ≥5: severe comorbidity.

### Anesthesia management protocol

Routine anesthesia monitoring included electrocardiogram (ECG), pulse oximetry (SpO_2_), end-tidal carbon dioxide, and systemic blood pressure monitoring. In addition to routine monitoring, processed EEG SedLine sedation monitoring (Masimo Corp., Irvine, CA, USA) was used in both groups.

The standard anesthesia induction protocol consisted of 1-2 mg/kg propofol, 1-2 μg/kg fentanyl, and 0.6 mg/kg rocuronium. Following induction, patients were intubated. General anesthesia was maintained with 1%-2% sevoflurane and 0.1-0.3 μg/kg/h remifentanil infusion.

### Intraoperative monitoring

Hemodynamic parameters, end-tidal minimum alveolar concentration, blood loss, and transfusions were recorded at 10-minute intervals in both groups. Additionally, in the ORi+SpO_2_ group, non-invasive ORi monitoring was performed.

### Neuro-monitoring

In both groups, intraoperative neuro-monitoring was conducted with the SEDLine Brain Function Monitor. This device is used to monitor four channels of raw EEG with symmetrical bifrontal electrodes. It provides separate displays for electromyogram, artifacts (such as patient motion), burst suppression ratio, and density spectral array (DSA). The SEDLine monitor estimates the depth of sedation from digital EEG waves using a proprietary algorithm, displayed as the patient state index (PSI), which ranges from 0 (isoelectric EEG suppression) to 100 (full wakefulness).

In both groups, PSI values were maintained between 25 and 50 to standardize the depth of anesthesia, reduce the risk of POD, avoid intraoperative awareness, and ensure adequate cerebral perfusion. Anesthesia depth was adjusted based on DSA and PSI values, which ensured optimal sedation levels while minimizing the risk of burst suppression.

### Hyperoxemia definition

ORi detects moderate hyperoxemia, which typically corresponds to partial pressure of oxygen (Pao_2_) levels between 100 and 200 mm Hg (13.3-26.7 kPa). It is a dimensionless index ranging from 0.00 (no oxygen reserve) to 1.00 (maximum oxygen reserve) measured using multi-wavelength pulse co-oximetry (17,18). While ORi does not directly measure PaO_2_, it provides a reliable alternative for monitoring oxygenation trends, particularly in settings where invasive measurements are impractical.

In this study, hyperoxemia was defined as a simultaneous occurrence of SpO_2_>98% and ORi ≥0.01. In the intervention group, FiO_2_ was dynamically adjusted based on real-time ORi and SpO_2_ values to prevent hyperoxemia, while in the control group, FiO_2_ adjustments were guided solely by SpO_2_ readings, as ORi values were recorded but not accessible to clinicians. In both groups, the initial FiO_2_ values were determined by the primary anesthesiologist based on his or her clinical judgment and routine practice.

### FiO_2_ titration

In the ORi+SpO_2_ group, ORi values were measured with the Rainbow and Radical-7® (both from Masimo Corp., Irvine, CA, USA). The target was to maintain 95%<SpO_2_≤98% and ORi at 0.00. FiO_2_ was adjusted based on ORi and SpO_2_ values, with changes made in 5% to 10% intervals to ensure that both parameters remained within the desired range.

### Postoperative intervention

Patients were evaluated in the postoperative recovery room with the Confusion Assessment Method for the ICU (CAM-ICU) and the Richmond Agitation-Sedation Scale (RASS). They were not discharged from the recovery unit until they achieved an Aldrete recovery score >9, a Visual Analog Scale pain score <5, and a Ramsey sedation score >5.

After leaving the recovery unit, patients were monitored either in the ICU or general wards. Their postoperative status was evaluated every 12 hours for two days with CAM/CAM-ICU and RASS.

### Outcomes and assessments

The primary outcome was the incidence of POD in elderly patients. POD was assessed using the CAM and its ICU-specific adaptation, CAM-ICU. The CAM tool is a widely used bedside screening instrument for detecting delirium in non-ICU patients who can verbally communicate, whereas CAM-ICU is a structured tool designed for ICU patients, including those who are non-verbal or mechanically ventilated, allowing for a rapid and accurate delirium diagnosis. The complementary use of these tools ensured standardized and reliable POD assessments, regardless of the patient's clinical setting.

POD was diagnosed based on at least one positive evaluation using either CAM or CAM-ICU during the 48-hour postoperative period. These assessments were made according to DSM-V criteria by an anesthesiologist blinded to group assignments. This structured approach aligns with clinical practice, as a single positive assessment was considered sufficient for POD diagnosis.

The secondary outcome was the incidence of intraoperative hyperoxemia episodes. Hyperoxemia occurrences were compared between groups to determine whether ORi-guided FiO_2_ titration effectively reduced hyperoxemia, potentially mediating its impact on POD incidence.

### Statistical methods

The sample size calculation, performed using OpenEpi 3.0 (*openepi.com*), determined the minimum sample size of 114 participants with an 80% confidence interval and a 5% margin of error.

Numerical data are reported as means ± standard deviations or medians (minimum-maximum), and categorical data as counts and percentages. For the primary outcome, we determined the overall proportion of patients in each group who experienced at least one POD episode across all repeated assessments. These proportions were compared with a Fisher exact test. To enhance interpretability, we also calculated the absolute difference between proportions with 95% confidence intervals (CIs) and the relative risk (RR). For the secondary outcome, the incidence of intraoperative hyperoxemia episodes was summarized descriptively. Since the number of hyperoxemia episodes was evidently lower in the ORi group, additional formal statistical testing was not deemed necessary. To compare the overall average FiO_2_ values between the groups, a student’s *t* test was used. The overall average FiO_2_ was calculated based on intraoperative FiO_2_ recorded at standardized time points throughout the procedure. Two independent numerical variables were compared with a *t* test or a Mann-Whitney U test. For comparisons involving more than two dependent groups, a Friedman test was performed, followed by a *post-hoc* analysis with a Dunn-Bonferroni test. For categorical variables, a Pearson χ^2^ test or a Fisher exact test was applied. A *P* value <0.05 was considered significant. The analysis was conducted with SPSS, version 25 (IBM Corp., Armonk, NY, USA).

## RESULTS

### Demographic and baseline characteristics

**Figure 1:**
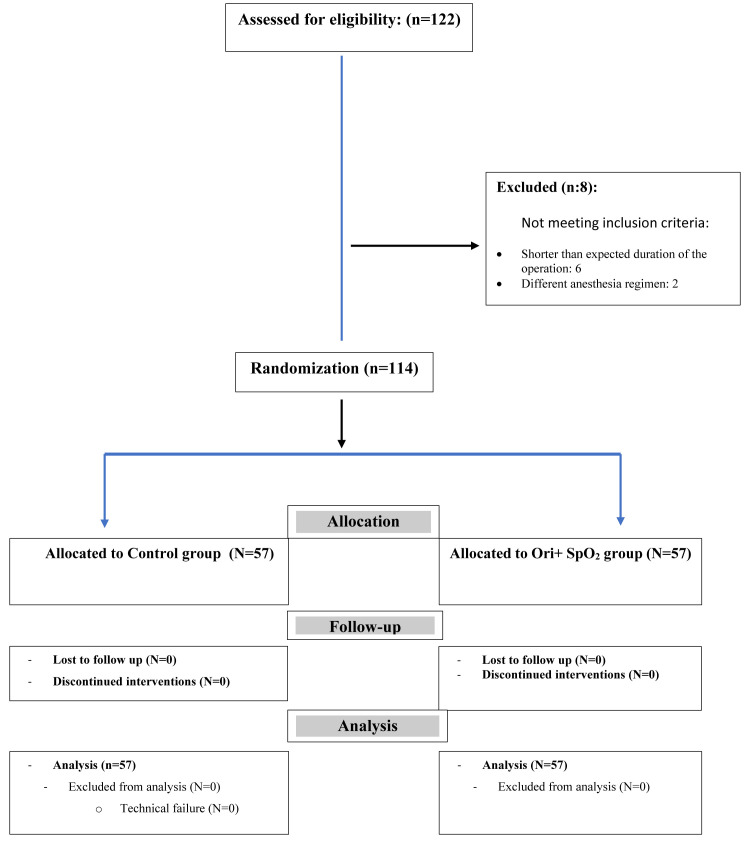
Study flowchart.

The study enrolled 114 patients (67.54% male) (Figure 1). There were significantly more men in the sample (*P* = 0.03). Smoking status was comparable between the groups, with 52.6% of smokers in the control group and 50.8% in the ORi+SpO_2_ (*P* = 0.78). The groups did not significantly differ in terms of age, ASA scores, preoperative MMSE scores, and Charlson Comorbidity scores (Table 1).

**Table 1:** Demographic data and preoperative evaluation

	Group	
	oxygen reserve index + pulse oximetry	control
	median (range)	median (range)
Age (years)	70 (68-73)	69 (67-72)
American Society of Anesthesiologists score	2 (2-3)	3 (2-3)
Charlson Comorbidity Score	3 (2-4)	3 (2-4)
	n (%)	n (%)
Sex		
male	30 (52.6)	28 (49.2)
female	27 (47.3)	29 (50.8)
Type of surgery		
abdominal	12 (21)	10 (17.5)
urogenital	18 (31)	14 (24.5)
gynecological	6 (10)	10 (17.5)
orthopedic	11(19.2)	9 (15.7)
otorhinolaryngology	10 (17.5)	14 (24.5)

### Intraoperative outcomes

The groups did not significantly differ in hemodynamic parameters and processed EEG values across all time points (Table 2). The overall incidence of POD was significantly lower in the ORi+SpO_2_ group (12.3%) than in the control group (42.1%) (*P* < 0.001, Table 3). The absolute difference in POD incidence was -29.8% (95% CI -44.8 to -13.9), and the relative risk reduction was 71% (RR 0.29, 95% CI 0.14-0.60). The prevalence of POD over time was lower in the ORi+SpO_2_ group than in the control group (Supplemental Table 1(Supplementary Table 1)).

**Table 2:** Hemodynamic parameters and processed EEG values across all time points

	Group		
Variable, mean ± standard deviation	control	oxygen reserve index + pulse oximetry	P*
Patient state index	38.65 ± 4.2	38.13 ± 4.3	0.524
Spectral edge frequency	13.14 ± 2.3	12.83 ± 2.5	0.502
End-tidal minimum alveolar concentration	1.52 ± 0.28	1.43 ± 0.2	0.071
Systolic blood pressure	112.41 ± 8.5	113.18 ± 8.9	0.640
Diastolic blood pressure	66.22 ± 7.5	69.21 ± 12.5	0.127
Heart rate	72.97 ± 12.8	71.80 ± 10.3	0.595
Duration of surgery (minutes)	158.8 ± 49.9	145.4 ± 40.6	0.260
Erythrocyte transfusion (units)	57	61	
Fresh frozen plasma transfusion (units)	57	63	

^*Student’s^ *^t^* ^test.^

**Table 3:** Postoperative delirium (POD) incidence in the control and intervention group

	No. (%) of patients				
Overall POD incidence	control	oxygen reserve index + pulse oximetry	Absolute difference (95% CI)	Relative risk (95% CI)	P*
No (-)	33 (57.9)	50 (87.7)	-29.8% (-44.8 to -13.9)	0.29 (0.14-0.60)	**<0.001**
Yes (+)	24 (42.1)	7 (12.3)			

^*Pearson χ2 test.^

Intraoperative hyperoxemia episodes, visually assessed across time points, were less frequent in the ORi+SpO_2_ group (Table 4).

**Table 4:** The number of patients with hyperoxemia across different time points

	No. (%) of patients in group	
Time (min)	control	oxygen reserve index + pulse oximetry
10	54 (94.7)	55 (96.5)
20	54 (94.7)	55 (96.5)
30	54 (94.7)	51 (89.5)
40	53 (93.0)	35 (61.4)
50	53 (93.0)	18 (31.6)
60	53 (93.0)	16 (28.1)
70	51 (89.5)	15 (26.3)
80	52 (91.2)	12 (21.1)
90	52 (91.2)	12 (21.1)
100	51 (89.5)	14 (24.6)
110	52 (91.2)	12 (21.1)
120	52 (91.2)	12 (21.1)

Although statistical tests were not applied, there was a clear difference in FiO_2_ titration patterns between the groups (Supplemental Figure 1(Supplementary Figure 1)). Detailed time-specific FiO_2_ measurements were consistent with the overall trend (data available upon request). Additionally, the overall average FiO_2_ levels reflected a generally lower oxygen exposure in the ORi+SpO_2_ group (Supplemental Table 2(Supplementary Table 2)).

When evaluating all patients with and without POD, FiO_2_ values were higher in patients with POD than in those without it. The overall trend suggests that patients with POD received higher FiO_2_ levels than those without POD (Supplemental Figure 2(Supplementary Figure 2))

## DISCUSSION

Our findings demonstrated that FiO_2_ adjustments guided by ORi significantly reduced the risk of POD. The incidence of POD was markedly lower in the ORi+SpO_2_ group than in the control group. These results suggest that tailored oxygenation strategies using non-invasive monitoring tools such as ORi could minimize postoperative neurocognitive complications.

Intraoperative oxygenation strategies play a crucial role in perioperative care, with most research focusing on the risks associated with hypoxemia rather than hyperoxemia (19-21). While ensuring adequate oxygenation is essential, excessive oxygen administration has been linked to atelectasis, hypercapnia, systemic vasoconstriction, and potential neurocognitive effects (11-13). Despite general recommendations to maintain FiO_2_ below 60% to mitigate these risks (20,21), the optimal intraoperative FiO_2_ levels remain debated.

Similar to the findings by Kupiec et al, our study supports a potential relationship between high intraoperative FiO_2_ levels and POD incidence in elderly patients (19). However, unlike previous studies that used Pao_2_ measurements, our study non-invasively assessed hyperoxemia using ORi as a surrogate marker.

Another key finding of this study is a higher incidence of intraoperative hyperoxemia in patients with POD. While our study did not establish a direct causal relationship between hyperoxemia and POD, it suggests that hyperoxemia may contribute to POD development. However, our findings need to be confirmed by advanced statistical modeling and prospective trials.

In our study, ORi-guided FiO_2_ titration effectively mitigated intraoperative hyperoxemia, which confirms that non-invasive real-time monitoring tools may optimize intraoperative oxygenation. Elevated FiO_2_ levels were associated with increased intraoperative hyperoxemia occurrences in the control group, which highlights the need for cautious oxygen management during surgery.

Several studies have examined the relationship between hyperoxemia and neurological outcomes. A meta-analysis showed that neurological prognosis worsened beyond PaO_2_ value of 154 mm Hg (13). Additionally, in a study involving 138 996 patients, lower intraoperative FiO_2_ levels were associated with a reduced mortality and fewer respiratory complications (22). Our findings align with this evidence, suggesting that careful FiO_2_ titration may improve perioperative outcomes in elderly patients.

Lin et al (16) found no significant difference in POD incidence between patients receiving 40% vs 80% FiO_2_. However, the observed lower rates of postoperative atelectasis in the lower FiO_2_ group highlight the complexity of oxygen management in elderly patients. Our study adds to this discussion by showing that while both hypoxemia and hyperoxemia pose risks, excessive oxygen exposure may contribute to increased POD incidence.

Many studies have explored the association between POD and postoperative cerebral oxygen saturation (SctO_2_) in elderly patients (23-27). While some researchers suggested that decreased SctO_2_ values correlated with POD, others found no significant difference in intraoperative SctO_2_ values between patients with and without POD (27). This indicates that factors beyond cerebral oxygenation, including intraoperative oxygenation strategies, may influence POD risk.

In our study, the groups did not significantly differ in terms of anesthesia depth, inhalation anesthetic doses, preoperative cognitive function, or intraoperative factors such as bleeding and transfusion amounts. This minimized confounding variables and allowed for a more focused examination of hyperoxemia’s role in POD development.

Although the ideal intraoperative FiO_2_ level remains controversial, some consensus guidelines suggest setting FiO_2_ to ≤0.4 once the airway is secured, with an aim to achieve normoxia while preventing hyperoxemia (20).

Our study has several limitations. First, the sample size was relatively small and restricted to a single-center setting, which may limit the generalizability of our findings to broader populations. Second, while the ORi was used as a non-invasive surrogate marker for oxygenation and to guide FiO_2_ titration, it does not directly measure PaO_2_ levels. This could restrict the understanding of the relationship between oxygenation status and POD. Third, the study focused on the immediate postoperative period without long-term follow-up assessments, which might provide insights into the lasting cognitive effects of intraoperative hyperoxemia. Lastly, POD is influenced by various factors, some of which are beyond the scope of this study. Further studies should employ advanced statistical methods to assess the multifactorial interactions in POD development.

In conclusion, adjusting FiO_2_ levels based on the ORi significantly reduced the incidence of POD in elderly patients undergoing elective surgeries. While intraoperative hyperoxemia was observed more frequently in the control group, the study design prevents us from concluding on the direct causal relationship between hyperoxemia and POD. Our findings suggest that non-invasive monitoring tools such as ORi can effectively guide oxygen delivery, reducing the risk of hyperoxemia and subsequently lowering POD incidence. These results underscore the importance of employing tailored oxygenation strategies to achieve adequate oxygen delivery while minimizing excessive administration.
