# Supplementary material for: Evaluating oxygen reserve index-guided oxygenation for the prevention of postoperative delirium in elderly patients: a randomized controlled trial
Source: Croat Med J. 2025 Feb;66(1):47–55. doi: 10.3325/cmj.2025.66.47 (PMC11947977; doi:10.3325/cmj.2025.66.47)
Supplement: Supplementary Table 1 [file CroatMedJ_66_s003.pdf]

Supplemental Table 1. Temporal Analysis of POD Incidence

		Control	Ori+Spo2
**CAM 12h**	No-delirium (-)	10(76.9)	27(100)
	Delirium (+)	3(23.1)	0
**CAM 24h**	No-delirium (-)	13(81.3)	28(100)
	Delirium (+)	3(18.8)	0
**CAM 36h**	No-delirium (-)	19(86.4)	35(97.2)
	Delirium (+)	3(13.6)	1(2.8)
**CAM 48h**	No-delirium (-)	19(86.4)	35(97.2)
	Delirium (+)	3(13.6)	1(2.8)
**CAM Postoperative**	No-delirium (-)	48(84.2)	51(91.1)
	Delirium (+)	9(15.8)	5(8.9)
**CAMICU 12h**	No-delirium (-)	28(63.6)	25(83.3)
	Delirium (+)	16(36.4)	5(16.7)
**CAMICU 24h**	No-delirium (-)	22(53.7)	23(79.3)
	Delirium (+)	19(46.3)	6(20.7)
**CAMICU 36h**	No-delirium (-)	16(45.7)	19(90.5)
	Delirium (+)	19(54.3)	2(9.5)
**CAMICU 48h**	No-delirium (-)	17(48.6)	19(86.4)
	Delirium (+)	18(51.4)	3(13.6)
