# Supplementary material for: Evaluating oxygen reserve index-guided oxygenation for the prevention of postoperative delirium in elderly patients: a randomized controlled trial
Source: Croat Med J. 2025 Feb;66(1):47–55. doi: 10.3325/cmj.2025.66.47 (PMC11947977; doi:10.3325/cmj.2025.66.47)
Supplement: Supplementary Table 2 [file CroatMedJ_66_s004.pdf]

**Supplemental Table 2. Comparison of FiO2 Levels Between Control and ORi+SpO2**

| Time/Groups | Control (Median [IQR]) | Ori+SpO2 (Median [IQR]) | p (Groups) |
|---|---|---|---|
| All-time average FİO2 | 45(28.75-60) | 28.75(25.42-41.67) | **<0.001** |
