# Supplementary figures and images for: Evaluating oxygen reserve index-guided oxygenation for the prevention of postoperative delirium in elderly patients: a randomized controlled trial
Source: Croat Med J. 2025 Feb;66(1):47–55. doi: 10.3325/cmj.2025.66.47 (PMC11947977; doi:10.3325/cmj.2025.66.47)

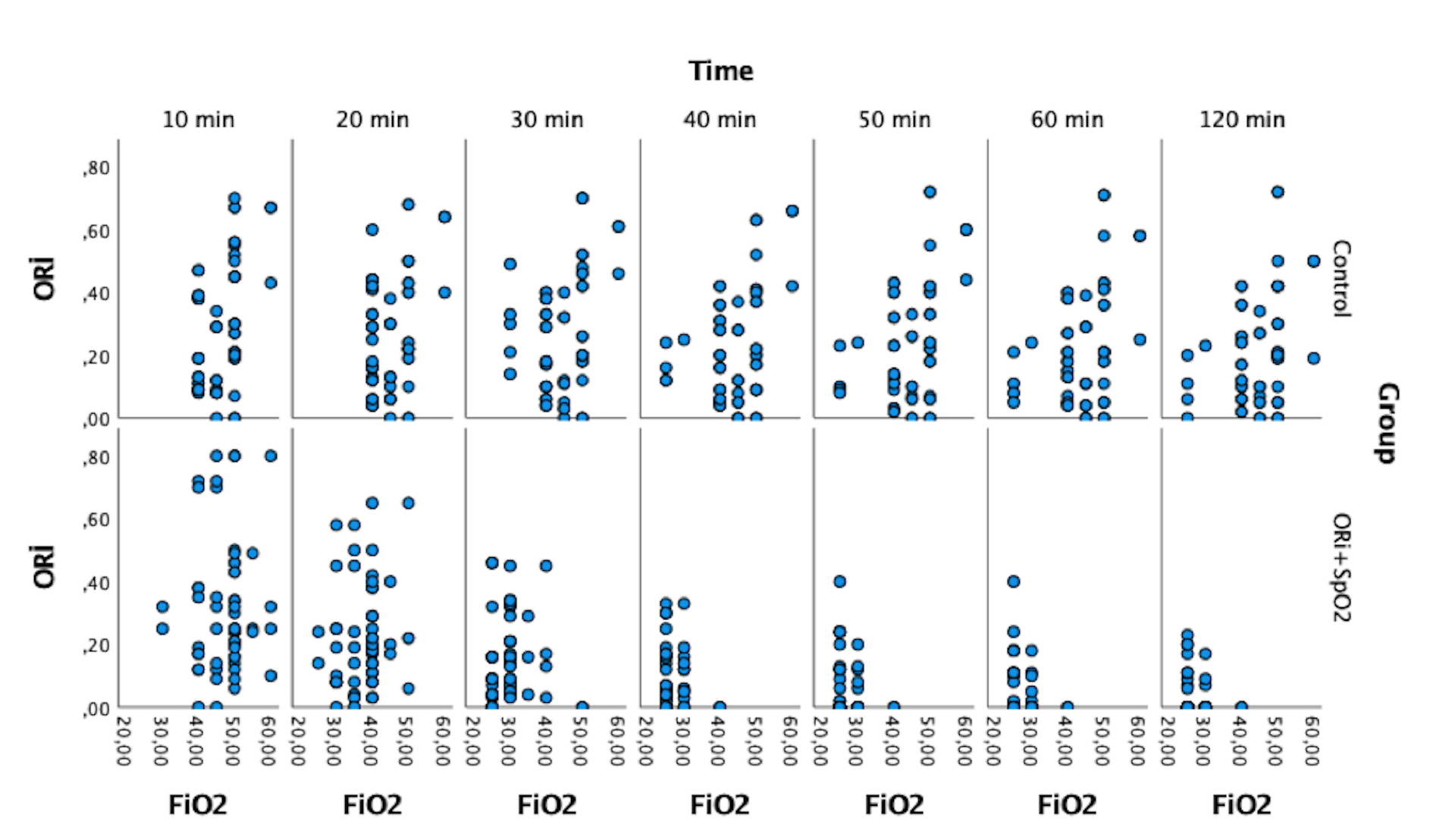

Supplement: Supplementary Figure 1 [file CroatMedJ_66_s005.jpg]

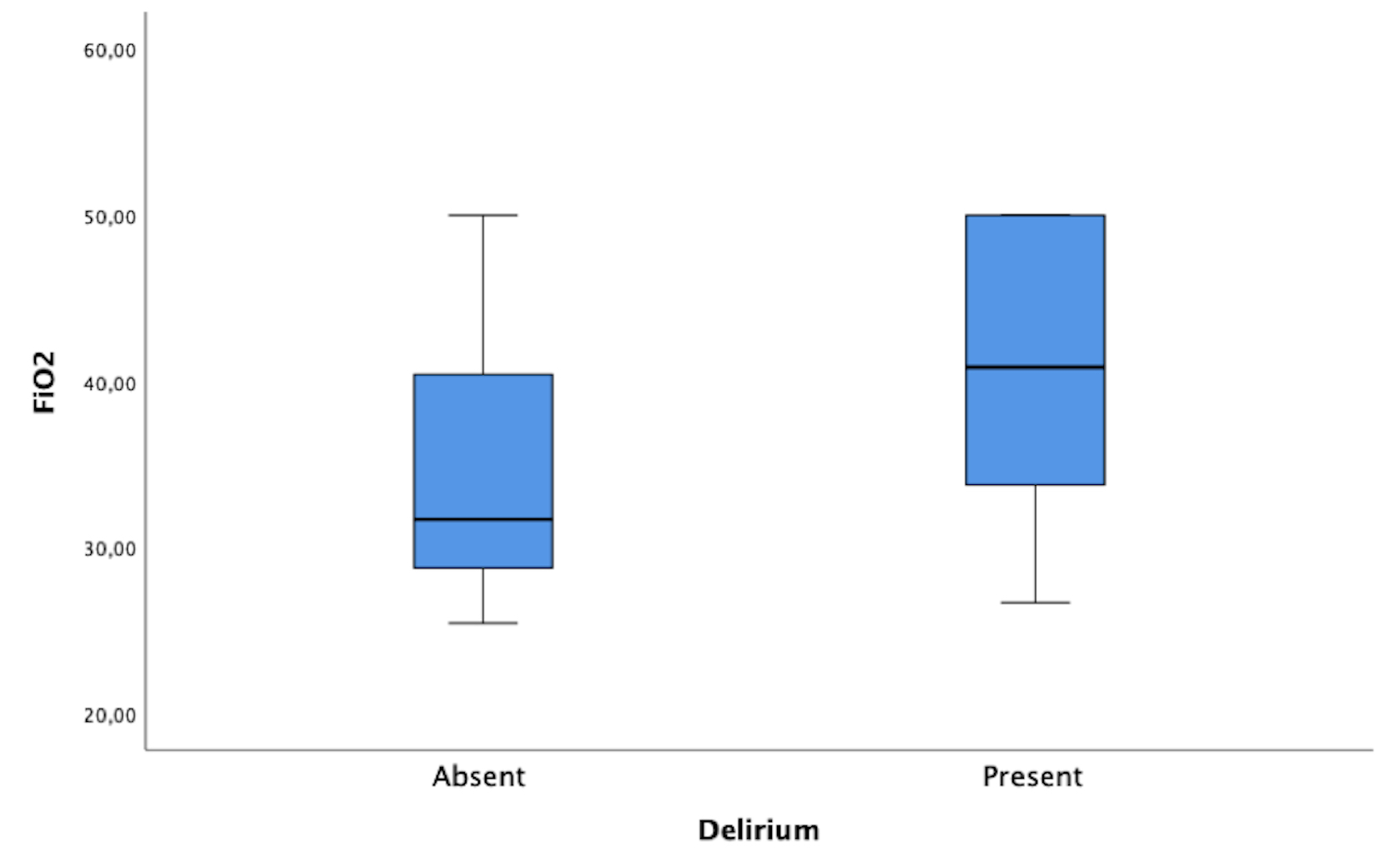

Supplement: Supplementary Figure 2 [file CroatMedJ_66_s006.jpg]
